# Naphthalene Detection in Air by Highly Sensitive TiO_2_ Sensor: Real Time Response to Concentration Changes Monitored by Simultaneous UV Spectrophotometry

**DOI:** 10.3390/s22197272

**Published:** 2022-09-26

**Authors:** Jorge H. Torres, Vincent A. Rosa, Patricia D. Barreto, Jose C. Barreto

**Affiliations:** 1 Department of Bioengineering, Florida Gulf Coast University, 10501 FGCU Blvd. South, Fort Myers, FL 33965, USA; 2 Department of Chemistry and Mathematics, Florida Gulf Coast University, Fort Myers, FL 33965, USA

**Keywords:** polycyclic aromatic hydrocarbons, air contaminant, semiconductor sensor, spectrophotometric monitoring

## Abstract

Volatile low-weight polycyclic aromatic hydrocarbons (PAHs) are known to be potentially toxic to humans and animals. Their detection in ambient air has been of great interest in recent years and various detection methods have been implemented. In this study, we used naphthalene as a basic model of such compounds and constructed our own version of a titanium oxide-based sensor system for its detection. The main goal of the study was to clearly demonstrate the effectiveness of this type of sensor, record its response under well-controlled conditions, and compare that response to concentration measurements made by the widely accepted spectrophotometric method. With that goal in mind, we recorded the sensor response while monitoring naphthalene vapor concentrations down to 95 nM as measured by spectrophotometry. Air flow over the sensor was passed continuously and sample measurements were made every 3 min for a period of up to 2 h. Over that period, several cycles of naphthalene contamination and cleaning were implemented and measurements were recorded. The relative humidity and temperature of the air being sampled were also monitored to assure no major variations occurred that could affect the measurements. The sensor showed high sensitivity and a reproducible response pattern to changes in naphthalene concentration. It could be easily “cleaned” of the compound in ten minutes by means of the application of UV light and the passing of fresh air. Pending testing with other volatile PAH, this type of sensor proves to be an effective and inexpensive way to detect naphthalene in air.

## 1. Introduction

Low-weight polycyclic aromatic hydrocarbons (PAHs) are volatile compounds produced by the burning of coal, oil, gas, and other industrial chemicals. As air contaminants, they are known to be potentially toxic to humans and animals [1]. For that reason there has been great interest in their detection in ambient air and various detection methods have been implemented over the years [2,3,4,5]. Of particular importance has been the development of inexpensive sensors that could be taken to the field, and be considered accurate, highly sensitive, easy to use and clean, and provide reproducible measurements. One type of sensor is based on the use of titanium oxide, a semiconductor material with photocatalytic properties [6,7,8]. Titanium-oxide-based sensors have been used to detect volatile organic molecules. A number of sensor variations and sensing methodology approaches have been reported for detection of those molecules [9,10,11,12,13,14,15,16,17,18,19]. Improved sensitivity and selectivity for specific organic compounds have been reported with particular titanium oxide structures like nanotubes [16,17] or combination (treatment) with other materials that become involved in the sensing process [18,19]. In our group we have developed our own version of a titanium oxide-based sensor system and have previously reported the capacity of the sensor to detect volatile hydrocarbons and its ability to self-cleaning in the presence of UV light [20]. Our methodology, though, remains as the most basic approach with a single thin layer of titanium oxide with no other material additions. This type of sensor is very simple, easy to make, and inexpensive. In that previous paper, which described detection of naphthalene as well as petroleum ether compounds, we pointed out the consistency and reproducibility of the sensor response to repeated cycles of contamination and cleaning. We indicated, on the other hand, a lack of sensor specificity as the sensor could detect both aromatic and aliphatic hydrocarbons, consistent with the fact that other researchers have used titanium-oxide sensors for detection of various organic molecules. Another important point that has to be made in regard to titanium oxide sensors is the impact of air relative humidity variations on sensor measurements during the actual detection process. Generally speaking, without specialized modifications with other materials, basic titanium oxide sensors for detection of contaminants in air are affected by the air relative humidity and temperature. These parameters need to be controlled and taken into account in order to properly detect a contaminant and make accurate concentration measurements. Titanium oxide is by itself a very good humidity sensor [21,22,23] and therefore an operational baseline must be defined under known and controlled air humidity. In this study, we specifically focused on the sensor’s ability to detect naphthalene as a basic model of PAH, carefully following, in real time, its response to changes in naphthalene concentration in air and its capacity to return to base line in the process of self-cleaning, all under well-controlled conditions of air relative humidity and temperature. We chose to use naphthalene as model of PAH for two main reasons: (a) it has a higher vapor pressure at room temperature compared to other PAHs (10.7 Pa at 298 K [24]) which allows it to sublime easier into the vapor form, and (b) it is less toxic than other PAH to handle by personnel in a non-chemically specialized lab. In our previous report on the response of our titanium-oxide sensor to detect hydrocarbons, including naphthalene [20], we used higher concentrations of the compound but those concentrations were not measured by any independent method during detection. An essential part of the study presented here was an accurate continuous real-time measurement of naphthalene concentration that could be related to the sensor response. That concentration was, therefore, simultaneously monitored by UV spectrophotometry. For a compound that well absorbs light on a particular wavelength, spectrophotometry is a widely accepted method to measure the compound’s concentration. In this regard, modern spectrophotometers are considered reliable in terms of sensitivity and accuracy. Other essential part of this study was the use of very low concentrations of naphthalene to better assess the sensitivity of this sensor. Other researchers have reported detection of naphthalene at very low concentrations, but using more elaborate methods [2,3]. We wanted to evaluate the sensitivity of our basic sensor down to naphthalene concentrations approaching the detection limit of the spectrophotometer.

## 2. Materials and Methods

### 2.1. Titanium-Oxide Sensor

**Figure 1:**
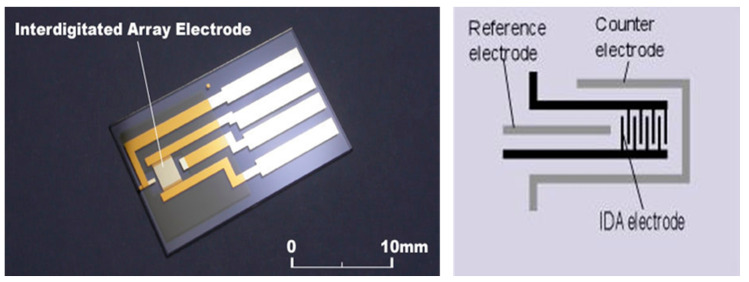
Picture (**left**) and diagram (**right**) of an Interdigitated Array Electrode (IDAE).

The sensor used in this study has already been described in a previous paper by our group [20]. It utilizes a platinum interdigitated array electrode coated by a titanium oxide layer whose thickness was carefully controlled. The coating layer was deposited by spraying the electrode with a solution of 10 mg/mL of commercial P-25 powder (Evonik, Essen, Germany) containing 5 nm titanium oxide particles. After drying and calcination at 600 °C for one hour, we obtained our final coated interdigitated array electrode (CoIDAE). Figure 1 below shows the array electrode before applying the coating.

### 2.2. Experimental Setup

**Figure 2:**
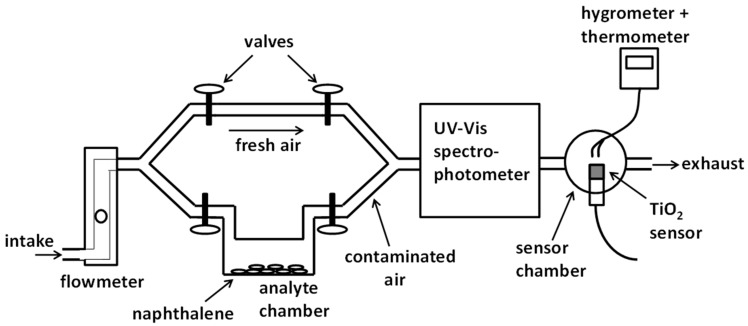
Diagram of the overall experimental apparatus with the major components. Not shown in this diagram is the UV-emitting LED array used to illuminate the TiO_2_ sensor. The LED array would be on top of the sensor out of the page (See pictures on next Figure 3).

A diagram of the overall experimental apparatus is shown in Figure 2. A vacuum pump (not shown) at the tubing output or exhaust created an air flow that passed through the whole apparatus. The air flow was controlled by the pump and measured by a rotameter-type flowmeter (Cole Parmer, Vernon Hill, IL, USA). Two lines of air passage were available, one line for clean or fresh air and another line for air going to a chamber containing the naphthalene analyte and becoming contaminated with naphthalene vapor. The naphthalene in the chamber was in the form of solid flakes that sublimated to naphthalene vapor which then was mixed with the air flowing through the chamber. Tubing for clean air was made of standard PFA plastic tubing. Tubing leaving the analyte chamber all the way to the sensor chamber was made of PTFE Teflon tubing to avoid naphthalene adhesion or wall permeation. We used an analyte chamber made of quartz to place the naphthalene flakes, although a regular glass chamber could have also been adequate. Either clean or naphthalene-contaminated air was made to pass through a 10 cm-long quartz chamber inside a spectrophotometer to measure naphthalene vapor concentration. Absorbance measurements were made at a wavelength of 265 nm corresponding to one of the peaks of the naphthalene absorption spectrum having its corresponding reported absorptivity value [25]. Measured absorbance values were easily converted to concentration values by simply using Beer’s law and the absorptivity of naphthalene. The spectrophotometer used was the Shimadzu UV-2450 UV-Vis spectrophotometer made by Shimadzu Corporation (with USA headquarters in Columbia, MD, USA). The tubing coming out of the spectrophotometer connected to the sensor chamber, a sealed glass chamber containing our own-made TiO_2_-based sensor and two small commercially available sensors to measure air relative humidity and temperature inside the chamber.

**Figure 3:**
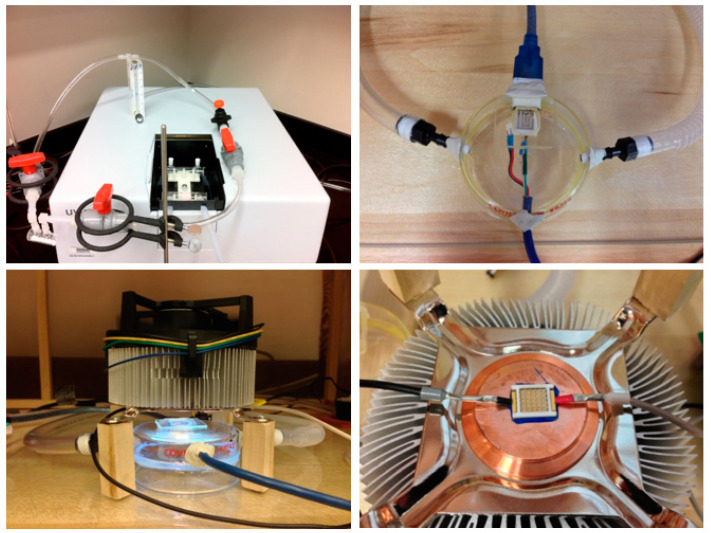
Pictures of: flowmeter, analyte chamber, and connections to the spectrophotometer (**top left**), sensor inside its glass chamber (**top right**), UV illumination (**bottom left**), and LED array with its cooling system (**bottom right**).

Pictures of flowmeter, analyte chamber with naphthalene flakes, spectrophotometer with corresponding tubing connections, sensor in its glass chamber, UV illumination of the sensor, and LED array used for the illumination are shown in Figure 3. The chamber contained the sensor made with the coated interdigitated array electrode (CoIDAE), the resistive polymer film and thermistor sensors for humidity and temperature measurements, respectively (top right picture of Figure 3). UV light was continuously applied to the TiO_2_ sensor which is known to absorb ultraviolet light and release electrons that are involved in photocatalytic reactions. Here, the TiO_2_ sensor was illuminated by a 5 × 5 UVA LED array with peak power at a wavelength of 375 nm, with a full width at half maximum (FWHM) of 12 nm, and a maximum total emitted power of 1250 mW over an aperture of 0.5 cm^2^ corresponding to 2500 mW/cm^2^ maximum emitted power density. The LED array illuminates the sensor from above (bottom left picture of Figure 3). The LED array was the Enfis Uno Tag Array UVA 375 nm manufactured by Enfis Limited (Swansea, UK). Since this LED array consumed 18 W of power, it generated significant amount of heat that had to be dissipated. For that heat dissipation we used an aluminum heat sink and a cooling fan normally used for cooling computer microprocessors. The LED array was placed 1.5 cm above the sensor. The sensor glass chamber had a quartz window on top to allow the passage of the UV light from the LED array to the sensor inside the chamber. The power density of the UVA light reaching the sensor was 50 mW/cm^2^ as measured by an optical UVA power meter placed at the same distance of the sensor with respect to the LED array. The LED array, with its heat sink and cooling fan, is shown in the bottom right picture of Figure 3. The sensor was connected to both an Agilent E3631A power supply (Keysight Technologies, Santa Rosa, CA, USA), which applied a designed voltage pulse, and a digital ammeter PXIe card (National Instruments, Austin, TX, USA) in a computer for real time collecting and recording of the sensor electrical current. The air passing through the system could be controlled by the vacuum pump at the exhaust end of the whole system. The pump to control the air was used as a vacuum pump at the exhaust instead of an air pump at the intake in order to avoid undesired contamination with pump oil products or other possible contaminants.

### 2.3. Experimental Procedure

A prepared TiO_2_-coated interdigitated array electrode (CoIDAE) was placed in the sensor chamber and pre-illuminated with UV light for 4 h to clean it of contaminants and to assure an optimized electrical response. While keeping the UV light on, a vacuum pump was turn on at the exhaust end to generate an air flow through the system whose input air was the laboratory ambient air considered clean of any naphthalene contamination. With the vacuum pump as air control, an air flow of 5 L/min was set for the intake. Using the control valves in the air pathways, either clean fresh air or naphthalene-contaminated air was sent to the spectrophotometer and the TiO_2_ sensor. Phases of clean and contaminated air were carried out in alternated cycles. The UV light was kept on all the time for all phases and cycles except when a sensor current measurement was made. The UV light could have a major effect on the TiO_2_-sensor electrical current measurement itself that needed to be investigated separately.

The concentration of naphthalene was varied by changing the amount of naphthalene flakes in the analyte chamber while maintaining the same air flow of 5 L/min. The electrical current through the CoIDAE in response to a 1 V voltage pulse was measured every 3 min. Each measurement produced a 10 s current recording which was distributed in time in the following manner: 2 s prior to the application of the voltage pulse in order to define the base line, 5 s corresponding to the 1V-pulse application, and 3 s after the pulse in order to follow the current recovery without voltage. Sensor current measurements were made in three consecutive phases according to the gas passing through the sensor’s chamber: (1) an initial baseline phase passing regular ambient air, (2) a phase during which air contaminated with naphthalene vapor was passed, and (3) a cleaning phase passing again regular ambient air. The cycle of naphthalene application and cleaning was repeated several times for up to 2 h. The UV light from the LED array was kept ON all the time throughout all the phases and cycles except for the 10 s of the sensor current measurement. This was done to avoid the effect of the UV light on the measurement itself that would have added another factor that complicated the analysis.

For every measurement of the sensor current response to the applied 1 V pulse, the peak current value was extracted from the recorded 10 s current signal and used to follow the sensor response as a function of time. Therefore, when plotting the response in time, each point in the graph corresponds to the peak current value at 3 min intervals. Simultaneously to the recording of the TiO_2_-sensor current signal, naphthalene concentration values were obtained from the passing-air absorbance measured by the spectrophotometer at a wavelength of 265 nm corresponding to one of the absorption peaks of naphthalene as it was indicated in the previous subsection. The naphthalene concentration was calculated from that measured absorbance, the length of the cuvette inside the spectrophotometer, and the naphthalene absorptivity value at that wavelength reported in the NIST WebBook [25]. The absorptivity value used was 3148 M^−1^cm^−1^. Absorbance measurements were made every minute with the spectrophotometer. Additionally, every minute, measurements of relative humidity and temperature of the air inside the sensor chamber were made with the resistive polymer film and thermistor sensors of the Traceable Hygrometer 4185 device from the company Traceable Products (Webster, TX, USA).

## 3. Results

**Figure 4:**
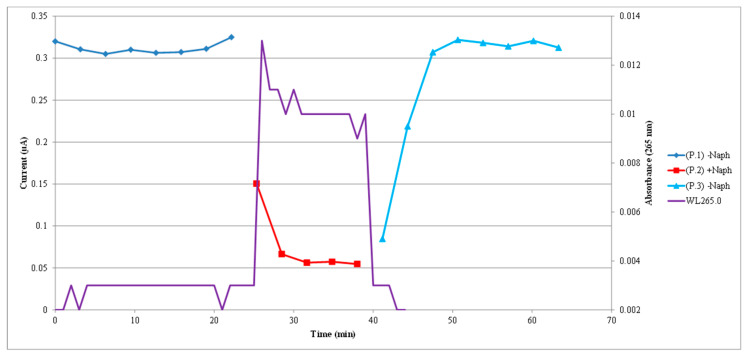
Typical sensor current response to one cycle of naphthalene contamination and cleaning. Micro-amp sensor current and absorbance measurements were made simultaneously. P1 corresponds to the initial fresh air introduction (baseline phase), P2 corresponds to naphthalene + air introduction (contamination phase), and P3 corresponds to the re-introduction of fresh air only (cleaning phase). The sensor current values can be read on the left-hand scale of the *y*-axis. The purple line (WL265.0) corresponds to the absorbance at 265 nm for naphthalene. Absorbance values can be read on the right-hand scale of the *y*-axis. An increase in absorbance corresponds to an increase in naphthalene concentration. Color lines with markers indicate sensor current measurements every 3 min. The purple line appears to be continuous because the spectrophotometer made absorbance measurements every minute. Sensor current decreased as naphthalene concentration increased.

The typical sensor response to one cycle of a naphthalene contamination followed by naphthalene removal with clean fresh air is shown in Figure 4. In the figure, sensor peak current values in response to a 1 V pulse applied to the sensor are plotted as a function of time with measurements taken every 3 min.

The darker blue line and points correspond to the initial clean baseline, the red line and points correspond to the naphthalene contamination phase, and the final light blue line and points correspond to the cleaning phase with clean fresh air. The electrical current values of the sensor response can be read on the left vertical axis scale. The purple more continuous line with no markers corresponds to the absorbance measurements made by the spectrophotometer and the absorbance numbers can be read on the right vertical axis scale. Those absorbance measurements were made every minute and translate, by using Beer’s law and the naphthalene absorptivity, into naphthalene concentration values as a function of time. The absorbance curve can be easily be correlated to the sensor current peak values. After converting absorbance to concentration values for the cycle presented in this figure, the naphthalene concentration in the contamination phase fluctuated between 381 nM (41 ppm by volume) and 318 nM (34 ppm). In response to that naphthalene concentration, the current through the sensor dropped from around 320 nA at baseline to 55 nA. In the cleaning phase with fresh air, the sensor current went back to about 320 nA, the initial baseline values.

**Figure 5:**
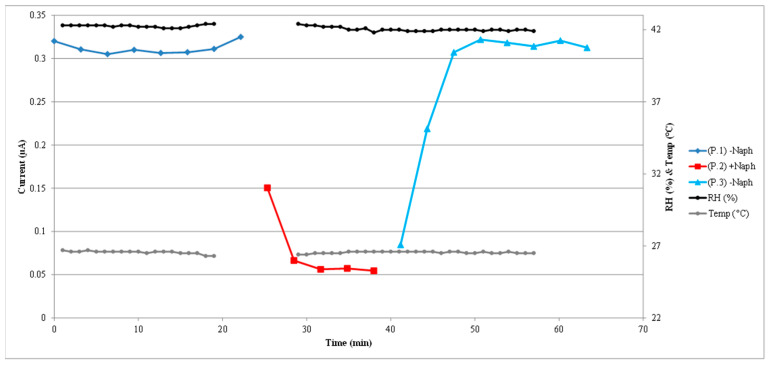
The same cycle of sensor response shown in Figure 4 is presented again here but now correlated in time with measured air relative humidity and temperature. The scale on the right vertical axis now corresponds to either the relative humidity or the temperature in degrees Celsius.

In Figure 5, the same cycle of sensor response shown in Figure 4 is presented again but now correlated in time with measured air relative humidity and temperature. The scale on the right vertical axis now corresponds to either the relative humidity or the temperature in degrees Celsius. The relative humidity and temperature were measured every minute. Although the recording of these parameters was interrupted for a short period, their values were very stable and consistent throughout the total one-hour recording.

**Figure 6:**
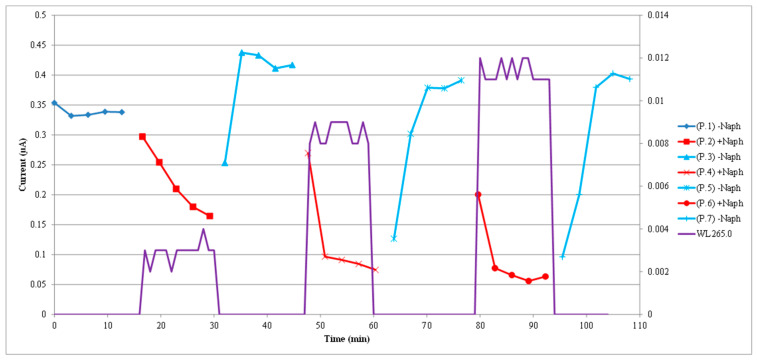
Three cycles with progressively increased naphthalene concentration in the respective contamination phases are shown. The last two cycles with higher naphthalene concentration show larger drop in sensor current. The red lines correspond to the naphthalene contamination phases, and the light blue lines correspond to the cleaning phases with the passing of fresh air. The more continuous purple line corresponds to the absorbance measured by the spectrophotometer. The scale of the right vertical axis corresponds to the absorbance.

Three cycles of naphthalene contamination and cleaning with fresh air are presented in Figure 6. These three cycles were run independently and separately from the run of the single cycle shown in Figure 4. In regard to the graph of Figure 6, the lines’ color format is the same as in Figure 4. The initial darker blue line corresponds to the initial baseline current, the red lines correspond to the naphthalene contamination phases, and the light blue lines correspond to the cleaning phases with the passing of clean fresh air. The more continuous purple line corresponds to the absorbance measured by the spectrophotometer. The scale of the right vertical axis corresponds to the absorbance. For the cycles presented here, the naphthalene concentrations based on the absorbance measured by the spectrophotometer were 95 nM (10 ppm), 286 nM (31 ppm), and 381 nM (41 ppm) for the respective contamination phases of the three sequential cycles. As the naphthalene concentration was progressively increased, the sensor peak electrical current progressively dropped during the contamination phases of the sequential cycles from the initial current at baseline. The sensor current values recovered during the corresponding cleaning phases of the three cycles. For these cleaning phases, the sensor current ended up higher than the current at the original baseline because of an air relative humidity drift that is shown in the next figure. Using the sensor current value before the contamination phase of each cycle, the following sequence of current drops occur with the presence of naphthalene: the current dropped from 340 nA to 164 nA (176 nA drop) for the first cycle, from 417 nA to 75 nA (342 nA drop) for the second cycle, and 391 nA to 56 nA (335 nA drop) for the third cycle. Although the third cycle had a higher concentration of naphthalene in the contamination phase than the second cycle, the current drops of the two cycles were similar because the drift in relative humidity affected the current measurements. The relative humidity is not shown in this Figure 6, but in the next figure. Overall, however, the sensor current decreases with increased naphthalene concentration and, for all three cycles presented here, the sensor current recovers completely after the cleaning phase with fresh air.

**Figure 7:**
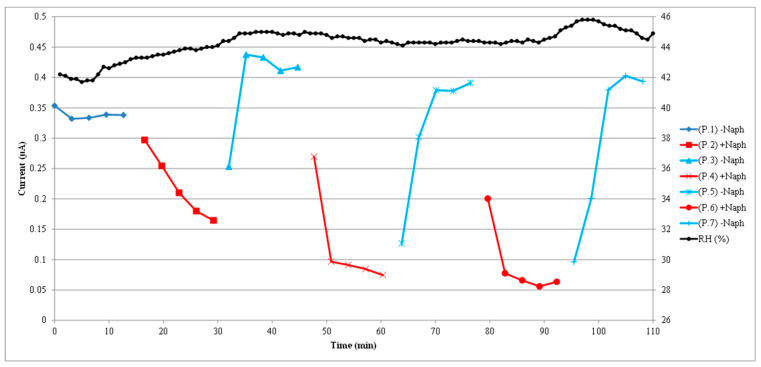
Same sensor current response presented in Figure 6, but now correlated to the air relative humidity. The scale of the right vertical axis corresponds to the humidity. The air relative humidity drifted upwards and fluctuated between 42% and 46%. Despite that drift, the trends remain for the sensor current response to drop with increased naphthalene concentration and to recover completely with cleaning with fresh air. The temperature remained relatively steady between 26 and 27 °C.

In Figure 7, the same sensor response shown in Figure 6 is presented, but now correlated in time with the air relative humidity. In this graph, the scale of the right vertical axis corresponds to the humidity. As it can be seen in the figure, the air relative humidity drifted upwards and slowly increased from 42% to 46%. Following the increase in air relative humidity, the clean air sensor current baseline also increased progressively. The effect seen in this figure brings up the need to compensate for any humidity variation when working with a titanium oxide sensor. The air temperature remained relatively stable between 26 and 27 °C and is not shown in the graph. Based on the results presented in Figure 6 and Figure 7, it is obvious that the sensor electrical response during the first cycle of the recorded sequence was affected by a variation in air relative humidity. For this first cycle, although the naphthalene concentration in the contamination phase was only 95 nM (10 ppm), the simultaneous increase in relative humidity that took place during this phase made the sensor current drop much slower when compared to the other cycles and the sensor response appeared sluggish. However, for the other two cycles in Figure 6 and Figure 7, as well as for the case of the single cycle shown in Figure 4 and Figure 5, most of the current drop occurred in the first 3 min of contamination.

These results show that, for all cases, complete recovery of the sensor electrical conductivity took place in 10 min after initiating the cleaning phase. Although not presented in the figures, we were able to reduce the naphthalene concentration to 30 nM (3.2 ppm), according to the absorbance measured by the spectrophotometer, and still elicit a well-observable response of the sensor. Lower concentrations were not tested, but we believe the sensor could detect concentrations down to 10 nM (about 1 ppm).

The graphs in the figures above are representatives of the sensor current response to variations in naphthalene concentrations. The same sensor response pattern repeated over and over again as the sensor was repeatedly exposed to similar cycles of naphthalene-contaminated air and fresh-cleaning air. Naphthalene concentrations varied, however, as we did not have exact control of the concentration with our experimental setup. Naphthalene concentration was changed by adding or removing naphthalene flakes from the analyte chamber. The resulting concentration was determined only after the spectrophotometer measurements were obtained and no attempt was made to set a specific concentration.

## 4. Discussion

Our research group has previously developed a version of a titanium oxide sensor for the detection of organic molecules in air. In this study we have used this sensor specifically for detection of naphthalene as the basic representative of the group of organic molecules known as volatile low-weight PAHs. Other researchers have also reported the use of a titanium oxide-type of sensor for detection of naphthalene as well as other organic molecules. Some of those sensors, however, although using titanium oxide, have been more elaborate versions utilizing nanotube structures or combining the titanium oxide with other materials [16,17,18,19]. We have used a simplified thin single layer of titanium oxide coating an interdigitated array electrode. This sensor is simple, easy to make, and inexpensive. As we have previously reported, the sensor has been easy to clean and reuse with a very reproducible response. That response had not been correlated to actual concentration measurements made independently and in real time. The main purpose of this study was to test the electrical response of this sensor under well-controlled conditions of air relative humidity and temperature and compare its response to naphthalene concentration changes that were monitored by the widely-accepted spectrophotometry method. Other goals of the study were to assess the sensor sensitivity to detect low naphthalene concentrations and to confirm its ability for self-cleaning and recovery after exposure to those low concentrations. The results showed that the sensor was very sensitive to naphthalene concentration changes and was capable of self-cleaning returning to previous values of electrical conductivity before the contamination. The sensor response to measured low concentrations followed the pattern we have previously observed and reported for higher concentrations not measured but implied by the larger amount of naphthalene used then. It is important to note that, as naphthalene concentration was increased, the sensor current decreased, but not proportionally. We expect that, as contaminant concentration increases, a form of exposure saturation relative to the surface area of the CoIDAE will be reached and further concentration increases of the contaminant will not result in any further change in the sensor current. Although the physicochemical mechanism of analyte interaction with titanium oxide is not fully understood, we had theorized, based on previous data we had obtained, that the binding of the analyte to the titanium oxide layer interferes with the free electron mobility in the layer. That explanation is further supported by the results of this study. Titanium oxide is a well-known semiconductor material that releases electrons when exposed to UV light. Those available free electrons establish a base current that, although small, is measurable in the range of nanoamps to microamps at applied voltages of 1 to 5 volts. For a thin layer of a few micrometers, like the one we are using in our sensor, the free electron availability and mobility in the layer will be interfered by the binding of an analyte like naphthalene filling physical gaps at the surface of the layer. Present water and water vapor as well as oxygen will take electrons from the titanium oxide and generate highly reactive radicals that react with the naphthalene initiating the process of its chemical breakdown. While naphthalene is continuously supplied and replenished, the steeling of free electrons continues and the current through the layer stays below baseline. Once naphthalene is not supplied any more in the passing air, the naphthalene bound to the surface of the titanium oxide is simply washed away or undergoes full chemical breakdown and the products washed out, leading to the recovery of the current through the layer. The titanium oxide layer must be very thin for the electrical current changes to be measurable. The layer thickness should be of the order of a few micrometers or, preferably, below one micrometer, since the detection mechanism is mainly a surface phenomenon. As the concentration of naphthalene increases, binding sites at the surface of the layer become saturated and no more naphthalene molecules can attach. When no more naphthalene can bind to the titanium oxide, no further reduction in the current occurs and the sensor response reaches a plateau. This is the scenario referred above as exposure saturation relative to the surface area of the CoIDAE. This study, that utilizes simultaneous real-time concentration measurements with spectrophotometry, corroborates the expected trend although full sensor response saturation was not observed within the time frame of our recordings.

Regarding the sensor sensitivity, we were able to elicit a sensor response at 30 nM (3.2 ppm), and we believe the sensor is capable of detecting concentrations of naphthalene down to 10 nM (about 1 ppm). On the tests whose graphs are presented in the results section, we have concentrations down to 95 nM (10 ppm). At the tested naphthalene concentrations, the spectrophotometric absorbance was significantly low and approaching the minimum threshold of detectability by the spectrophotometer. The titanium-oxide sensor current response was still strong and could potentially be present even at naphthalene concentrations below the detection capabilities of the spectrophotometer. Detection of naphthalene at these concentrations indicates that this sensor is highly sensitive. The sensitivity of our sensor is not too far away from the sensitivity of 0.5 ppm reported by Girschikofsky et al. for their highly sensitive optical sensor for naphthalene [2], although their sensor is far more elaborate to build. Certainly, our sensor cannot match the sensitivity of 1 ppb reported by Leidinger et al. for their WO_3_ sensor prepared by pulse laser deposition [3], which is the most sensitive naphthalene sensor reported in the literature. Compared to these more elaborate sensors, our sensor still has very good sensitivity with the advantage of low cost and simplicity of construction. Regarding the form of response of our sensor in this study, it responded to an increase in naphthalene concentration with a decrease in electrical conductance that followed well the changes in naphthalene concentration measured by the spectrophotometer. The sensor responded to naphthalene contamination with a drop in electrical conductance which occurred mostly in the first 3 min and was able to recover completely its original baseline conductance in 10 min after initiating the cleaning phase. However, the results also confirmed the sensitivity of this type of sensor to air relative humidity changes and reminded us of the necessity to control that humidity or compensate for it. Figure 7 in the results section clearly shows the drifting upwards of the clean air sensor current baseline as the relative humidity drifts slowly from 42 to 46%. In this regard, it must be mention that, although not presented here, we have performed independent humidity tests with this sensor that showed an approximate linear trend. For practical use of this sensor in the field, calibration with spectrophotometry can be performed and compensation for humidity variations can be incorporated in that calibration. Although we only tested naphthalene as a model of volatile low-weight PAHs and did not test other more potentially toxic PAHs in our laboratory, we believe this titanium oxide-based sensor constitutes a simple and relatively inexpensive method to detect this type of organic compounds in the field. The sensor is capable of self-cleaning and its response is very reproducible. We did not present more graphs of the sensor response as a function of time, since the response pattern was the same and very reproducible in separate recordings taken at different times. The response patterns in time were very similar to those we presented for the same sensor in our previous paper, although in that study no naphthalene concentration measurements were made (but expected to be much higher). Additional graphs here would not provide additional information. Other separate recordings have comparable concentrations but not the same as those in the graphs above because we did not have control to set exact specific concentrations with our experimental setup. In future work we plan to collect a large number of samples of sensor current values in response to specific naphthalene concentrations in order to better evaluate the sensor reproducibility and define a calibration curve that can be used for measurements in the field. This study was more focused on the response pattern with changes in concentration measured in real time.

As indicated in the introduction, this basic single-layer titanium-oxide sensor has the drawback of its lack of specificity. Beside naphthalene, this sensor also responds strongly to the presence of other types of organic compounds. Using a different experimental apparatus, we previously reported strong sensor response to both aromatic and aliphatic hydrocarbon compounds [20]. Other researchers have also reported the use of titanium oxide for detection of a good variety of organic molecules. To achieve some selectivity they have combined titanium oxide with other materials or made other significant modifications [16,17,18,19]. We are currently investigating ways to obtain some form of sensor selectivity to low-weight PAHs using naphthalene as a model and comparing to the sensor response to other organic compounds. For naphthalene in particular some results have been encouraging but further studies have to be carried out. The ultimate goal is to build an inexpensive portable device that could be taken to the field to detect the presence of volatile PAHs in ambient air and measure their concentrations. The components that we are currently using with this titanium-oxide sensor can definitely be put together in a compact portable device. The study presented here can be easily followed by a systematic refined study of sensor response to a series of naphthalene concentrations. A calibration curve of sensor current as a function of naphthalene concentration corrected for ambient air relative humidity can be obtained. The issue that remains as a main challenge is the issue of specificity. In the present form and with the current detection methodology, this sensor would detect a number of volatile hydrocarbons that may be present as contaminants in air tested in the field. Currently, therefore, the sensor works great as a general detector of volatile hydrocarbons and could be used right now in the field with that purpose in mind. However, additional work seeking specificity is needed before use in the field for the particular detection of PAHs. We continue our work in this regard.

## 5. Conclusions

Evaluation of a titanium oxide sensor response to the presence of naphthalene in air was performed in this study under well-controlled conditions of air relative humidity and temperature and with the monitoring of naphthalene concentrations with spectrophotometry. The results confirmed the ability of the sensor to detect naphthalene with high sensitivity. It followed a reproducible response pattern with changes in naphthalene concentration and reaffirmed its ability for self-cleaning in the presence of UV light. Ambient air relative humidity must be taken into account when using the sensor for naphthalene concentration measurements. Ways to improve sensor specificity need to continue being explored.

## Data Availability

Not applicable.
